# Post-treatment plasma EBV-DNA positivity predicts early relapse and poor prognosis for patients with extranodal NK/T cell lymphoma in the era of asparaginase

**DOI:** 10.18632/oncotarget.4505

**Published:** 2015-07-03

**Authors:** Liang Wang, Hua Wang, Jing-hua Wang, Zhong-jun Xia, Yue Lu, Hui-qiang Huang, Wen-qi Jiang, Yu-jing Zhang

**Affiliations:** ^1^ State Key Laboratory of Oncology in South China, Collaborative Innovation Center for Cancer Medicine, Guangzhou, Guangdong, 510060, People's Republic of China; ^2^ Department of Hematologic Oncology, Sun Yat-sen University Cancer Center, Guangzhou, Guangdong, 510060, People's Republic of China; ^3^ Department of Medical Oncology, Sun Yat-sen University Cancer Center, Guangzhou, Guangdong, 510060, People's Republic of China; ^4^ Department of Radiation Oncology, Sun Yat-sen University Cancer Center, Guangzhou, Guangdong, 510060, People's Republic of China

**Keywords:** extranodal NK/T cell lymphoma, prognosis, Epstein-Barr virus, asparaginase, minimal residual disease

## Abstract

Circulating Epstein-Barr virus (EBV) DNA is a biomarker of EBV-associated malignancies. Its prognostic value in early stage NK/T-cell lymphoma (NKTCL) in the era of asparaginase was investigated. 68 patients were treated with a median of 4 cycles of asparaginase-based chemotherapy followed by a median of 54.6Gy (range 50–60Gy) radiation. The amount of EBV-DNA was prospectively measured in both pretreatment and post-treatment plasma samples by real-time quantitative PCR. At the end of treatment, complete response (CR) rate was 79.4%, and overall response rate (ORR) was 88.2%. Patients with negative pretreatment EBV-DNA had a higher CR rate (96.0% vs. 69.8%, *p* = 0.023). The 3-year progression-free survival (PFS) rate and overall survival (OS) rate was 71% and 83%, respectively. In multivariate survival analysis, post-treatment EBV-DNA positivity and treatment response (non-CR) were prognostic factors for both worse PFS and OS (*p* < 0.05). Local tumor invasion was also a prognostic factor for worse OS (*p* = 0.010). In patients with CR, post-treatment EBV-DNA positivity correlated with inferior PFS and OS (both *p* < 0.0001). In patients with positive pretreatment EBV-DNA, negative post-treatment EBV-DNA correlated with better PFS and OS (both *p* < 0.0001). These findings indicate that post-treatment EBV-DNA positivity can predict early relapse and poor prognosis for patients with early stage NKTCL in the era of asparaginase, and may be used as an indicator of minimal residual disease.

## INTRODUCTION

Extranodal NK/T-cell lymphoma (NKTCL) is a distinct subtype of non-Hodgkin lymphoma (NHL), and closely associated with Epstein-Barr virus (EBV) infection [1]. This disease is relatively rare in the United States and Europe but much more common in EBV-endemic areas, such as East Asia, Southeast Asia, and Central and South America [2]. In recent years, many cancer centers have adopted the combination of chemotherapy and radiotherapy (RT) (primary chemotherapy followed by RT or concurrent chemoradiotherapy) for localized disease to reduce the relapse rate [3–5]. Due to overexpression of multi-drug resistance (MDR) gene in NKTCL cells [6], anthracycline-based chemotherapy regimens are no longer the standard of care for NKTCL, and with increasing evidence asparaginase-based regimens are now frontline treatments [5, 7–9].

Circulating cell-free EBV DNA can be detected in plasma from patients with EBV-associated neoplasm [10–12]. Lei et al. [13] demonstrated a close correlation in NKTCL patients between clinical outcomes and plasma EBV-DNA levels (*N* = 18). Recently, Wang et al. [14] explored the prognostic value of plasma EBV-DNA levels in a relatively homogenous cohort of patients with early-stage NKTCL who received primary radiotherapy (*N* = 69) and found that both the pretreatment and post-treatment EBV-DNA level can serve as a valuable biomarker of tumor load and prognostic factors. However, all patients in the study reported by Wang et al. [14] received upfront radiotherapy, and half the patients received CHOP or CHOP-like chemotherapy regimens, which are now considered inappropriate for NKTCL patients [15]. Thus, in the era of asparaginase, whether plasma EBV-DNA levels can still retain their prognostic value or not remains to be investigated. Kwong et al. [16] investigated the role of EBV-DNA in patients treated with SMILE (dexamethasone, methotrexate, ifosfamide, L-asparaginase and etoposide), and found that post-treatment EBV-DNA levels are a prognostic factor for overall survival (OS). However, the toxicities related with SMILE treatment are severe [8] and rarely used in China. Wang et al. [5] and Lin et al. [4] demonstrated that GELOX (gemcitabine, oxaliplatin, and asparaginase) and CHOPL (cyclophosphamide, adriamycin, vincristine, prednisone, and asparaginase) were well tolerated and had great activity in the treatment of early stage NKTCL.

In this prospective observational study, we explored the correlation between plasma EBV-DNA levels and clinical features (such as response rate and survival) in early-stage NKTCL patients treated with upfront asparaginase-based chemotherapy (GELOX or CHOPL) followed by radiotherapy.

## RESULTS

### Patients’ characteristics and pretreatment EBV-DNA level

The patients’ characteristics are listed in Table 1. In our cohort of 68 patients, the median age was 47 years old (range 13–79), with 16 patients (23.5%) being older than 60 years old. Nearly half patients had stage I disease, and most patients (77.9%) had normal lactate dehydrogenase (LDH) level. 83.8% of patients were categorized to low-risk group (IPI = 0–1) according to International Prognostic Index (IPI) system. All patients had primary tumor site located in the upper aerodigestive tract, with 17 patients (25%) having extranasal disease. As is shown in Table 1, 43 patients (63.2%) in our cohort had positive pretreatment EBV-DNA. More patients with positive pretreatment EBV-DNA had elevated LDH (27.9% vs.12.0%), B symptoms (51.2% vs. 28.0%), ECOG performance status score >1 (27.9% vs. 16.0%), and higher IPI score (20.9% vs. 8.0%), but all differences were not significant.

**Table 1:** Patients’ characteristics and pretreatment EBV-DNA level

Parameters		Pretreatment EBV-DNA negative (*n* = 25)(%)	Pretreatment EBV-DNA positive (*n* = 43)(%)	*P* value
Gender	Male	16 (64.0%)	25 (58.1%)	0.826
	Female	9 (36.0%)	18 (41.9%)	
Age	>60	3 (12.0%)	13 (30.2%)	0.158
	= < 60	22 (88.0%)	30 (69.8%)	
Stage	I	14 (56.0%)	21 (48.8%)	0.750
	II	11 (44.0%)	22 (51.2%)	
LDH	Elevated	3 (12.0%)	12 (27.9%)	0.222
	Normal	22 (88.0%)	31 (72.1%)	
Local tumor invasion	Yes	15 (60.0%)	26 (60.5%)	1.000
	No	10 (40.0%)	17 (39.5%)	
ECOG PS score	0–1	21 (84.0%)	31 (72.1%)	0.412
	> = 2	4 (16.0%)	12 (27.9%)	
B symptoms	Yes	7 (28.0%)	22 (51.2%)	0.108
	No	18 (72.0%)	21 (48.8%)	
IPI score	0–1	23 (92.0%)	34 (79.1%)	0.194
	> = 2	2 (8.0%)	9 (20.9%)	
Primary tumor site	Nasal	22 (88.0%)	29 (67.4%)	0.110
	Extranasal	3 (12.0%)	14 (32.6%)	
Treatment response	CR	24 (96.0%)	30 (69.8%)	0.023
	Non-CR	1 (4.0%)	13 (30.2%)	

Abbreviations: LDH, lactate dehydrogenase; ECOG, Eastern Cooperative Oncology Group; PS, performance status; IPI, International Prognostic Index; CR, complete response.

### Treatment response and post-treatment EBV-DNA level

All patients received a median of 4 cycles (range 2–6) of asparaginase-based chemotherapy followed by a median of 54.6Gy (range 50–60Gy) RT. At the end of treatment, 54 patients (79.4%) got complete response (CR), 6 patients (8.8%) got partial response (PR), resulting in an overall response rate (ORR) of 88.2%. As is shown in Table 1, patients with negative pretreatment EBV-DNA had significantly higher CR rate (96.0% vs. 69.8%, *p* = 0.023). Post-treatment EBV-DNA was positive in 15 patients (22.1%), of whom the treatment response was evaluated as CR in 10 patients (66.7%), PR in 1 patient (6.7%), and disease progression (PD) in the remaining 4 patients (26.7%).

### Long-term survival outcomes and survival analysis

At a median follow-up time of 32 months (range 2–76), 17 patients had disease progression or relapse at a median of 5.3 months (1–28.3), of whom 10 patients died of tumor progression at a median of 9 months (3.4–25.2). The 3-year progression-free survival (PFS) rate and overall survival (OS) rate was 71% and 83%, respectively.

**Figure 1:**
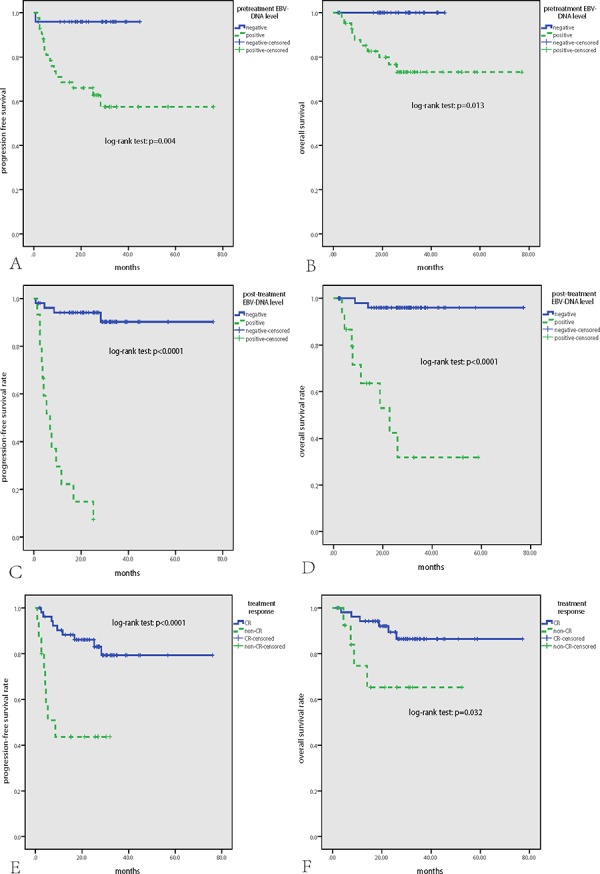
Survival analysis in the whole cohort of 68 patients with NKTCL Positive pretreatment EBV-DNA level **A, B.** positive post-treatment EBV-DNA level **C, D.** and no CR after treatment **E, F.** were significantly associated with inferior PFS and OS.

As is demonstrated in Figure 1 and Table 2, in univariate survival analysis, stage (II), pretreatment EBV-DNA level (positive), post-treatment EBV-DNA level (positive), and treatment response (non-CR) significantly correlated with both inferior PFS and OS (*p* < 0.05), while ECOG PS score (>1) was associated with poor PFS (*p* = 0.025) and local tumor invasion was associated with poor OS (*p* = 0.022). In multivariate survival analysis that including all parameters found to be significant in univariate analysis, it was found that post-treatment EBV-DNA level (positive) and treatment response (non-CR) were significantly prognostic factors for both worse PFS and OS (*p* < 0.05), and local tumor invasion was also a significantly prognostic factor for worse OS (*p* = 0.010).

**Table 2:** Univariate and multivariate survival analysis

Parameters	PFS			OS		
	Univariate analysis	Multivariate analysis		Univariate analysis	Multivariate analysis	
	*p* value	HR (95% CI)	*p* value	*p* value	HR (95% CI)	*p* value
Age (>60)	0.959			0.913		
Stage (II)	0.005	-	-	0.019	-	-
B symptoms (yes)	0.404			0.339		
LDH (elevated)	0.429			0.549		
LTI (yes)	0.479			0.022	22.172 (2.096–234.526)	0.010
Primary tumor site (extranasal)	0.685			0.720		
ECOG PS score (>1)	0.025	-	-	0.113		
Pre-treatment EBV-DNA level (positive)	0.004	-	-	0.013	-	-
Post-treatment EBV-DNA level (positive)	<0.0001	42.744 (10.613–172.151)	<0.0001	<0.0001	46.236 (7.503–284.913)	<0.0001
Treatment response (non-CR)	<0.0001	8.015 (2.471–26.000)	0.001	0.032	7.357 (1.295–41.793)	0.024

Abbreviations: PFS, progression free survival; OS, overall survival; CI, confidence interval; LDH, lactate dehydrogenase; LTI, local tumor invasion; ECOG, Eastern Cooperative Oncology Group; PS, performance status; CR, complete response.

**Figure 2:**
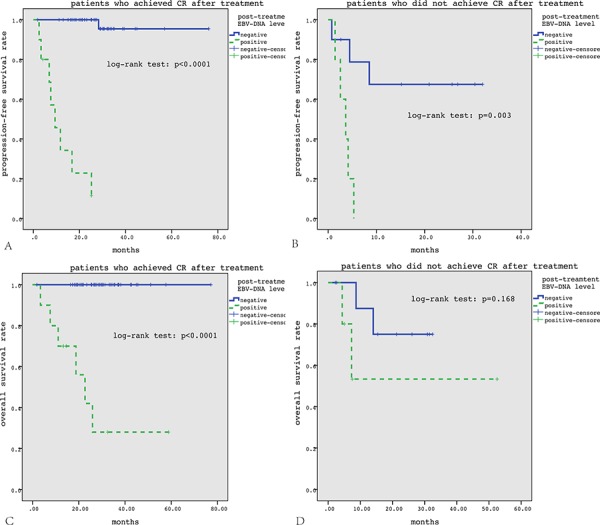
Subgroup survival analysis according to treatment response In patients who got CR after treatment, positive post-treatment EBV-DNA level correlated with significantly inferior PFS and OS **A, C.** In patients who did not get CR after treatment, positive post-treatment EBV-DNA level correlated with significantly inferior PFS but not OS **B, D.**

**Figure 3:**
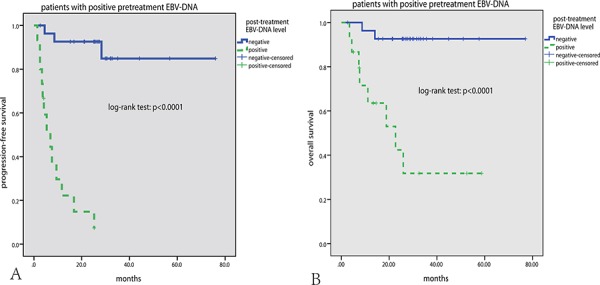
Subgroup survival analysis according to pretreatment EBV-DNA level In patients with positive pretreatment EBV-DNA level, negative post-treatment EBV-DNA level correlated with significantly superior PFS and OS **A, B.**

In subgroup analysis, of the 54 patients who got CR, post-treatment EBV-DNA level was positive in 10 patients, and these patients had significantly inferior PFS and OS than those with negative post-treatment EBV-DNA level (both *p* < 0.0001) (Figure 2-A, 2-C). Of 14 patients who did not get CR, post-treatment EBV-DNA level was negative in 10 patients, and these patients had significantly better PFS than those with positive post-treatment EBV-DNA level (*p* = 0.003) (Figure 2-B, 2-D). Of the 43 patients who had positive pretreatment EBV-DNA, post-treatment EBV-DNA level was negative in 28 patients (65.1%), and these patients had significantly better PFS and OS than those with positive post-treatment EBV-DNA level (both *p* < 0.0001) (Figure 3).

## DISCUSSION

In this study of NKTCL patients treated with an asparaginase-based method, we confirmed that pretreatment EBV-DNA levels correlated with a decreased treatment response, and post-treatment EBV-DNA levels were a prognostic factor for both PFS and OS. Moreover, we proposed that post-treatment EBV-DNA levels could be used as a biomarker of minimal residual disease (MRD) for NKTCL.

The lymphomagenesis of NKTCL is closely associated with EBV infection [1, 17]. Previous studies found that pretreatment EBV-DNA levels can reflect the tumor load [13, 14, 18]. In our study, it appeared that more patients with positive pretreatment EBV-DNA had higher levels of tumor load, such as elevated LDH level, B symptoms and higher IPI scores, however, all these differences were not significant. The discrepancies between our result and previous results may be influenced by the following factors: (1) only early stage patients were included in our study and differences in tumor load were sometimes not obvious between stage I and II disease; (2) in this study, we chose 0 copy/mL as the cutoff value for the pretreatment EBV-DNA level, and we used chi-square analysis for the correlation between EBV-DNA levels and clinical features. In contrast, most previous studies [14, 16] used Mann-Whitney tests to compare the median level of EBV-DNA between different groups. These different methods may contribute to the different results; (3) small sample sizes, including our study. Thus, large cohort studies or meta-analysis should be performed to validate these results.

In recent years, studies find that high expression of MDR gene in NKTCL cells may lead to primary resistance to anthracyclines-based chemotherapy [6] and that asparaginase-based regimens have an impressive response rate in NKTCL patients [4, 5, 7, 8]. In this study, after asparaginase-based chemotherapy followed by RT, the 3-year PFS and OS rate was 71% and 83%, respectively, which seems better than our retrospective results of CHOP followed by RT (3-year PFS and OS rate was 43% and 54%, respectively) [7, 15] This finding needs to be validated in prospective randomized clinical trials. However, no biomarkers are defined yet to predict the response rate of asparaginase-based treatment. In our study, patients with positive pretreatment EBV-DNA had a lower CR rate. Similarly, Kwong et al. [16] demonstrated that higher levels of pretreatment EBV-DNA predicted poor response rates after chemotherapy with SMILE regimen, indicating that the pretreatment EBV-DNA level may be a good candidate for the prediction of response after asparaginase-based treatment. If validated in future perspective clinical trials, patients with high pretreatment EBV-DNA levels may benefit from more intensive treatments or novel drugs other than asparaginase-based therapy.

Most previous studies searched for valuable prognostic factors in NKTCL patients [15, 17, 19–26]. IPI, Korean prognostic score (KPI), local tumor invasion, and treatment response were confirmed by different studies as independent prognostic factors in the era of pre-asparaginase. Whether these factors remain to be of value requires further investigation Kwong et al. [16] found that in patients treated with SMILE chemotherapy, both IPI and KPI lost their prognostic values when the EBV-DNA level was included in the final Cox regression model. Similar work was published when our paper was submitted for publication [27], finding that post-treatment EBV-DNA positivity correlates with PFS and OS in patients with NKTCL. In our present study, most elements of IPI and KPI (such as age, stage, LDH level, B symptoms, ECOG performance status) were found to be uncorrelated with prognosis, indicating the poor prognosis brought by these factors might be overcome by asparaginase-based therapy. Our study also found that pretreatment EBV-DNA levels were not an independent prognostic factor for both PFS and OS and that the final treatment response was much more valuable in predicting the prognosis. This is consistent with the results of a previous study [16] where presentation EBV DNA levels did not have an impact on survival independently, implying that the sensitivity of the tumor to asparaginase-based therapy, rather than its load, might be a more important indicator of survival. Most importantly, post-treatment EBV-DNA positivity was the most valuable independent prognostic factor for PFS and OS in patients treated with asparaginase-based therapy and this result was similar with previous studies [14, 16, 18]. Post-treatment EBV-DNA positivity may indicate MRD, which has been widely used as a prognostic factor for various hematologic malignancies. In most patients, a positive MRD may reflect less sensitivity to asparaginase-based therapy and an increase in disease relapse. In patients who had CR with positive post-treatment EBV-DNA levels, their PFS was shorter than those with a negative EBV-DNA level. Furthermore, some patients did not have CR after treatment, however they also had negative post-treatment EBV-DNA levels, possibly indicating an indolent nature of these patients, and their PFS was relatively better. Finally, for patients with high levels of pretreatment EBV-DNA, a negative post-treatment EBV-DNA level indicates quick tumor reduction. This implies that the disease was highly sensitive to asparaginase-based therapy, thus bestowing these patients with superior treatment outcomes.

In conclusion, we find that post-treatment EBV-DNA positivity in plasma can predict early relapse and poor prognosis for patients with early stage NKTCL in the era of asparaginase, and may be used as an indicator of minimal residual disease.

## MATERIALS AND METHODS

### Patients

Between January 2008 to December 2014, 68 patients diagnosed as stage IE/IIE NKTCL in Sun Yat-sen University Cancer Center were included in this study. The pretreatment and post-treatment plasma samples were prospectively collected. This study was approved by the Institutional Review Board (IRB) of Sun Yat-sen University Cancer Center. All patients provided written informed consent for the collection and publication of their medical information at the first visit to our center. The inclusion criteria for this study were as follows: (1) confirmed diagnosis of NKTCL according to the World Health Organization (WHO) classification for lymphomas [28], and *in situ* hybridization for EBV encoded RNA (EBER) should be positive for all patients; (2) primary tumor site located in the upper aerodigestive tract; (3) stage IE and IIE disease; (4) complete clinical information and follow-up data; (5) no prior therapy for NKTCL.

All patients received upfront asparaginase-based chemotherapy, such as CHOPL [4] and GELOX [5]. After a median of 4 cycles (range 2–6) of chemotherapy, patients were given a median of 54.6Gy (range 50–60Gy) RT using intensity-modulated radiotherapy (IMRT) or three dimension conformal radiotherapy [5]. We defined the clinical target volume of limited stage IE disease as the bilateral nasal cavity, bilateral ethmoid sinuses, and ipsilateral maxillary sinus; and the clinical target volume extended to involved tissues for patients who had extensive stage IE disease. For patients who had stage IIE disease, the clinical target volume also included the bilateral cervical lymph node area. Tumor response was assessed after every 2 cycles of chemotherapy or before and after RT on the basis of standardized response criteria for non-Hodgkin lymphoma [29].

### Patient samples and DNA preparation

Plasma samples were collected from patients within one week before initiation of chemotherapy and one month after completion of RT. All plasma samples were frozen at −80°C before further processing. Total plasma cell-free DNA was isolated using the QIAamp Blood Mini Kit (QIAgen, Inc., Valencia, CA, USA) acc ording to the “blood and body fluid protocol” as recommended by the manufacturer.

### EBV-DNA quantification

The real-time quantitative PCR system was developed for plasma EBV DNA detection toward the BamHI-W region as previously reported [30, 31]. The system consisted of the amplification primers W-44F (5′-AGT CTC TGC CTC AGG GCA-3′) and W-119R (5′-ACA GAG GGC CTG TCC ACCG-3′) and the dual-labeled fluorescent probe W-67T (5′-[FAM]CAC TGT CTG TAA AGT CCA GCC TCC[TAMRA]-3′). EBV-negative healthy volunteers were used as negative controls, and a no template control was run on each plate as a blank control. The results were expressed asthe number of copies of EBV per milliliter (mL) of plasma.

### Statistical analysis

The correlation between pretreatment EBV-DNA levels and clinical features was analyzed using the Mann-Whitney test. The comparison of qualitative data was performed using the chi-square analysis. According to the method we used for EBV-DNA quantification, the detection limit was defined to be 1000 copies/mL (any level between 0–1000 copies/mL was positive, but the value was not accurate), thus we chose 0 copy/mL as the cutoff value for both pretreatment and post-treatment EBV-DNA level. Progression-free survival (PFS) was calculated from the date of diagnosis to the date of disease progression or death and was censored at the date of the last follow-up visit. Overall survival (OS) was calculated from the date of diagnosis to the date of death from any cause and was censored at the date of the last follow-up visit. Survival analysis was performed using the Kaplan-Meier method, and comparisons were calculated using the log-rank test. Multivariate analysis was used to estimate the prognostic impact of different variables in OS and PFS using the Cox regression model. Differences between the results from comparative tests were considered significant if the two-sided *p* value was <0.05. All statistical analyses were performed using PASW Statistics 18.0 software (Apache Software Foundation, Forest Hill, Md).
